# Antimicrobial Activity of Nanoparticle Calcium Hydroxide against *Enterococcus Faecalis*: An *In Vitro* Study

**Published:** 2014-12-24

**Authors:** Omid Dianat, Sara Saedi, Majid Kazem, Mostafa Alam

**Affiliations:** a * Iranian Center for Endodontic Research, Department of Endodontics, Dental School, Shahid Beheshti University of Medical Sciences, Tehran, Iran; *; b *Research Institute of Dental Sciences, Shahid Beheshti University of Medical Sciences, Tehran, Iran; *; c *Department of Oral and Maxillofacial Surgery, Dental School, Shahid Beheshti University of Medical Sciences, Tehran, Iran*

**Keywords:** Calcium Hydroxide, *Enterococcus Faecalis*, Intracanal Medicament, Nanoparticle

## Abstract

**Introduction:** *Enterococcus faecalis* (*E. faecalis*) has the ability to invade the dentinal tubules and resist high pH levels. As a result, calcium hydroxide (CH) is not much effective on this bacterium. In theory, nanoparticle calcium hydroxide (NCH) has smaller size and high surface area that enables it to penetrate into the deeper layers of dentin and be more effective on *E. faecalis.* This *in vitro* study was designed to compare the antimicrobial activity of NCH and CH against *E. faecalis*. **Methods and Materials:** The antimicrobial activity of NCH against *E. faecalis* was evaluated by two independent tests: the minimum inhibitory concentration (MIC) of intracanal medicament and agar diffusion test (ADT). The efficiency of the medicament in dentinal tubules was evaluated on 23 human tooth blocks that were inoculated with *E. faecalis*. The tooth blocks were assigned to one control group (saline irrigation) and two experimental groups receiving CH and NCH as intracanal medication. The optical density in each group was assessed with spectrophotometer after collecting samples from dentin depths of 0, 200 and 400 µm. Data were analyzed by SPSS software ANOVA, Kruskal-Wallis and Dunnett’s test. **Results: **The MIC for NCH was 1/4 of the MIC for CH. NCH with distilled water (DW) produced the greatest inhibition zone in agar diffusion test. NCH had greater antimicrobial activity in dentin samples from depths of 200 and 400 µm compared to CH. **Conclusion: **The antimicrobial activity of NCH was superior to CH in culture medium. In dentinal tubules the efficacy of NCH was again better than CH on the 200- and 400-µm samples.

## Introduction

Incomplete disinfection of the complex root canal system may result in treatment failures and persistent apical periodontitis [1]. Previous studies proved the bacterial penetration into the depths of 300 to 1500 µm in dentinal tubules [2]. The bacteria in these depths would remain inaccessible for conventional irrigants, medicaments and sealers [3, 4]. Nanoparticles are microscopic particles with dimension less than 100 nm. These nano-sized particles are different in properties such as active surface area, chemical and biological reactivity [5]. Various nanoparticles are getting popular in dentistry and medicine as antibacterial agents. The higher surface to volume ratio and charge density of these materials, result in their greater interaction with the environment and thus causes a higher antibacterial activity [6-8].

Calcium hydroxide (CH) is widely used as an intracanal medicament [9-11]. This bactericidal medicament has a high pH level (~12). In order to be effective, the hydroxyl ions (OH^-^) in CH should diffuse into the dentinal tubules and accessory canals where the bacteria are harbored. The release of these ions induces an alkalizing effect and destroys the cellular membranes and protein structures. CH also dissolves the remaining tissue debris. It has the ability to promote an osteogenic environment and prevent root resorption [12-15]. Although CH is the most common intracanal medicament used in endodontics, it cannot be considered as a universal medication against all specimens harbored in root canals [16]. Bystrom *et al.* [17] showed that *Enterococcus faecalis (E. Faecalis)* is the most resistant strain against CH. In addition, previous studies reported that CH is not effective against the *E. Faecalis* for over 10 days of medication [18].

*E. faecalis* is a facultative gram positive bacterium, isolated mostly from the root-filled teeth with chronic apical periodontitis. There are different reports on the prevalence of this strain of bacteria in retreatment cases. Culture assays demonstrated almost 37% prevalence in root-filled teeth with apical periodontitis, while polymerase chain reaction (PCR) techniques reported the frequency of *E. faecalis* between 12 to 77% [19-21]. *E. faecalis* has the ability to invade the dentinal tubules and adhere to collagen and form a biofilm on dentin. The endodontic medicaments, including CH, cannot access the *E. faecalis* due to its penetration to the depths of 300 µm in dentinal tubules. *E. faecalis* can also survive high pH levels varying from 9 to 11 [22]. An *in vitro* study in culture tubes showed that a 10.5 to 11 pH level could only retard *E. faecalis* growth; whereas, no bacterial growth was seen at pH levels of 11.5 and greater [23]. The buffering capacity of dentin prevents CH from producing the required pH level with lethal effects on *E. faecalis* when it reaches the deeper dentinal tubules [24, 25]. Therefore, the nanoparticle calcium hydroxide (NCH) was manufactured with a modification to the method described by Roy and Bhattacharya [26].

The aim of this *in vitro* study was to compare the antimicrobial efficacy of NCH against *E. faecalis* to that of CH by measuring the minimum inhibitory concentration (MIC) and agar diffusion test (ADT) in dentin models from different depths.

## Materials and Methods

*E. faecalis* (ATCC 29212), was obtained from the Microbiology Department of Shahid Beheshti University of Medical Sciences. The antimicrobial activity of NCH against *E. faecalis* was evaluated in 3 stages and compared with conventional CH.

***Stage 1: Determination of minimum inhibitory concentration (MIC)***

The MIC of medicament which can inhibit the microbial growth, was assessed in both groups: NCH (Institute of Polymer Research, Karaj, Iran) and CH (Merck, Darmstadt, Germany). The following procedure was similar for both groups. 

In each group100 mg of powder was dissolved in 0.5 cc of normal saline and then saline was added until the solution reached the final volume of 1 cc. Therefore, a concentration of 100 mg/mL was obtained in each group. Then 0.5 cc of the brain-heart infusion (BHI) broth (Difco Laboratories, Detroit, MI, USA) was poured in 10 sterile tubes and 0.5 cc of the previously prepared primary solution was transferred to the first tube to reach half of the primary concentration. Afterwards, 0.5 cc of the solution in each tube was removed and poured into the next one to obtain dilute solutions with 1/4, 1/8, 1/16, 1/32, 1/64, 1/128, 1/256, 1/512 and 1/1024 concentration of the primary solution.

*E. faecalis* was cultured in tryptic soy broth (TSB) (Difco Laboratories, Detroit, Mich., USA) and stored at 37^°^C for 48 h. To achieve a bacterial suspension with a concentration of 0.5 McFarland containing 1.5×108 cells/mL the microbial cells were resuspended with saline. Then 0.01 mL of this suspension was inoculated in tubes and incubated for 24 h in 37^°^C. The results were confirmed by transferring the samples with an inoculating loop on sterile bile esculin agar (BBE) culture plates.

The culture results were evaluated with light microscopy and the results were recorded. To ensure the accuracy of the results, each concentration of the medicament was examined twice and if needed the test was repeated for each subgroup.

***Stage 2: Agar diffusion test (ADT)***

The antimicrobial effects of CH and NCH in combination with normal saline and 0.2% chlorhexidine (CHX) (Iran Najo Pharmaceutical Co., Tehran, Iran) were assessed as follows: 0.1 mL of microbial suspension of *E. faecalis* with 0.5 McFarland scale was inoculated in 5 Petri plates containing BBE. The plates were randomly assigned to 5 groups including four experimental groups and one control group with no medication. Four groups of medications were defined as below (distilled water=DW): CH+DW, CH+0.2% CHX, NCH+DW and NCH+0.2% CHX.

The medicaments were prepared in a creamy consistency by mixing the powders with 1 cc of liquid. Blank discs were placed in the experimental plates under complete sterile conditions. All plates were stored at 37^°^C for 24 h. Afterwards the diameter of the inhibition zones around each wall was measured and recorded in mm. This procedure was repeated for three times for each group and the mean value of the inhibition zones was reported.

***Stage 3: Dentin sample***

***Tooth block preparation: ***The methodology of this part was a modification of the method offered by Haapasalo and Orstavik dentin model [4]. Twenty three single-rooted newly extracted human teeth were collected. The specimens were kept in 5.25% NaOCl for 24 h to remove the tissue remnants. The roots were cut at 2 to 3-mm distances from the CEJ, with a diamond disk to obtain 14 to 16-mm sections. The root canals were instrumented with hand K-files (Dentsply Maillefer, Ballaigues, Switzerland) and ProTaper rotary system.

To remove the smear layer the samples were soaked in an ultrasonic bath (Biosonic UC 300, Whaledent, Switzerland) of 17% EDTA (Merck Co., Darmstadt, Germany) for 5 min and in 5.25% NaOCl for another 5 min [10, 27]. The samples were stored in DW for 24 h. Each sample was placed in a microtube containing 2 mL of TSB and was autoclaved in 121˚C for 30 min. To make assure the accurate sterilization procedure, one microtube was selected randomly and the TBS solution was incubated for 24 h in 37˚C to assess microbial growth.

***E. Faecalis inoculation: ***One mL of the TBS in each microtube was removed by an insulin syringe. One mL of *E. faecalis* suspension with optical density of 0.5 McFarland was injected by an insulin syringe inside each sample. After 24 h of incubation in 37˚C, 3 samples were randomly selected to ensure the microbial inoculation. The samples remained in the incubator for 21 days to ensure the bacterial penetration into the dentinal tubules. The TBS solution in each microtube was replaced every 3 days.

The bacteria were transferred by means of #30 paper cones (Ariadent, Tehran, Iran) from tubes containing 2 mL of BHI culture media. The tubes were incubated for 24 h in 37˚C and the optical density of each sample was measured by spectrophotometer at 625 nm wavelength.

***Intracanal medication procedure***

The samples were randomly assigned to 3 groups. DW was added to 1 g of powder to prepare the pastes. The groups included: group 1, *E. faecalis*+CH (*n*=8); group 2, *E. faecalis*+NCH (*n*=8) and group 3 (control), *E. faecalis*+DW (*n*=3). Eight root canals were filled with the NCH and 8 canals with CH. DW was delivered into 3 canals by syringes. All the samples were incubated again in 37˚C for 7 days.

***Assessment of microbial content in different dentin depth***

After this period, each canal was irrigated with 10 mm of normal saline. A sterile paper cone was used to transfer the microbial content of the intracanal wall surface to 2 mL of BHI media. To assess the microbial content of different depths, dentin layers were shaved by sizes 2 and 4 Gates Glidden drills from the depths of 200 and 400 µm, respectively. The samples were all transferred to 2 mL of BHI media and incubated for another 24 h in 37˚C. The optical density of each tube was evaluated with spectrophotometer at wavelength of 625 nm, by comparing the optical density of each tube with the standard tube of 0.5 McFarland.

***Statistical analysis***

Data were analyzed by SPSS software (version 16.0, SPSS, Chicago, IL, USA). Data in each group were compared by the ANOVA and Kruskal-Wallis tests. Also the Dunnett’s test was performed to compare the results between two groups. The level of significance was set at 0.05.

## Results

***MIC***

Both medicaments were able to efficiently inhibit the growth of *E. faecalis*, though the amount of MIC between the groups was different. The evaluations revealed that NCH was more effective than conventional CH and acted in smaller amounts, as the 1/16 dilute tube (6.25 mg/mL) showed no microbial growth in NCH group, while in the CH group the 1/4 dilute solution (25 mg/mL) did not allow microbial growth.

**Table 1:** Mean (SD) of agar diffusion test results (ZOI=zone of inhibition, DW=distilled water, CHX=chlorhexidine, NCH=nanoparticle calcium hydroxide)

**Medication**	**CH+DW**	**CH+CHX **	**NCH+DW**	**NCH+CHX **
**ZOI (mm)**	10.5 (1.6)	11.2 (2.1)	14.2 (0.7)	11.9 (0.8)

***ADT results***

Based on the results of Kruskal-Wallis test, the mean size of ZOI in the experimental groups were significantly different after 24 h (*P*<0.05). The highest efficacy belonged to NCH+DW, while the lowest efficacy was reported for conventional CH+DW. The efficacy of medicaments from the highest to the lowest in descending order was: NCH+DW>NCH+CHX>CH+CHX>CH+DW. The efficacy of NCH+CHX and CH+CHX was almost similar. The means of ZOI in each group are demonstrated in Table 1.

***Optical density***

ANOVA showed no significant differences in optical density of groups before medication (*P*=0.21). The evaluated optical densities on the 7th day after treatment and in the control group are presented in Table 2. When evaluating the optical density of samples removed from the dentin surface by Dunnett’s analytical test, there was no significant difference between the control group and NCH (*P*=0.14) and between the control group and CH (*P*=0.23). In the depth of 200 µm, NCH showed more efficacy and Dunnett’s test revealed a significant difference between NCH group and the control group (*P*<0.05), while there was no significant difference between the CH and the control group (*P*=0.18). The results of ANOVA test showed that the optical density of samples collected from the depth of 400 µm were significantly different between groups (*P*<0.05). The antimicrobial efficacy of both experimental groups were higher than the control group as the Dunnett’s test showed a significant difference between the control group and CH and between the control group and NCH (*P*<0.05).

## Discussion

In this *in vitro *study the antimicrobial efficacy of NCH and CH against *E. faecalis* was compared by means of two independent tests including MIC and ADT. The antimicrobial activity of nano-sized CH particles was higher compared to that of conventional CH. 

Human dentin blocks were used as previously described by Haapasalo and Orstavik [4]. The activity of medicaments is weaker *in* *vivo* compared to *in* *vitro*. Using the dentin model enlightened that although CH rapidly killed the bacteria in the test tubes, only partial disinfection can be achieved in the surface wall of the root canal and CH was relatively ineffective [4]. The buffering capacity of dentin was proved by another study in which the effectiveness of CH paste against *E. faecalis* combined with different concentrations of dentin powder was evaluated [28].

**Table 2:** Mean (SD) of optical density after 7 days of treatment in different depths (NCH=nanoparticle calcium hydroxide)

**Tubular depth (µm)**	**CH **	**NCH**	**Control**
**0 **	0.87 (0.12)	0.80 (0.08)	1.17 (0.21)
**200**	0.82 (0.08)	0.70 (0.12)	1.14 (0.07)
**400 **	0.76 (0.14)	0.68 (0.09)	1.11 (0.14)

In similar studies which used human dentin blocks CH showed a weaker antimicrobial activity against *E. faecalis* than other medicaments such as triple antibiotic paste [29, 30].

In this study, the MIC assessment revealed that NCH inhibited *E. faecalis* in lower doses than the conventional form of CH. In this study 25 mg/mL of CH inhibited the growth of *E. faecalis* which is higher than the amount of MIC reported by Pallotta *et al.* (16 mg/mL) [31].

The other *in vitro* test used in this study was ADT which is a widely used test with reproducible results. However, the medication activity may be influenced by its dissolution capability and the buffering capacity of the culture media, and this phenomenon is critical for antimicrobial efficacy of CH which is pH-dependent. In this study the ZOI was measured only after 24 h, however smaller ZOIs were reported in previous studies at longer durations [22]. Previous studies on different formulations of CH have controversial results. Some of these studies showed that CH combined with CHX was more effective on *E. faecalis* than CH mixed with DW in ADT [13, 14, 32]. Sukawat and Srisuwan [12] declared that there is no difference between the antimicrobial activity of CH powder mixed with CHX or DW. The results of the present study confirmed this fact.

We hypothesized that the nanoparticles of CH would penetrate dentinal tubules deeper and at a greater concentration than CH and therefore NCH would be more effective than CH against *E. faecalis*. To evaluate this hypothesis, dentin block models were used and the bacterial concentrations in different dentin depths (200 and 400 µm) were assessed by spectroscopy after 7 days of medication. There was no difference between the antimicrobial efficacy of conventional and nanoparticle medicaments on the surface. In the dentinal depths of 200 µm and 400 µm the NCH was more effective against *E. faecalis. *These results can prove the hypothesis; however, there should be a larger sample size to evaluate the efficacy of NCH in further studies. In this study the samples were compared quantitatively by spectroscopy; but for a more accurate evaluation of bacterial presence in the dentinal tubules following medication, PCR test is recommended which can detect 10 to 10² cells and is more sensitive and reliable than culturing methods [33]. The medication period in this study was 7 days considering the fact that CH paste needs 7 days to reach the optimum pH level [34]. It was also reported that *E. faecalis* needs over 10 days of medication with CH to be influenced [35]. Prolonged periods of medication can be considered in further studies.

*E. faecalis* resists high pH levels. It maintains a pH level by the buffering capacity of its cytoplasm. It also has a proton pump in which it provides additional homeostasis. However, studies have reported that this microorganism cannot resist pH levels over 11.5 [23, 35]. Therefore CH needs to penetrate dentinal tubules in enough concentrations to reach a pH of ≥11.5. The buffering capacity of dentin should also be considered in this regard [36, 37]. 

After preparing a 1% solution of NCH, the pH level of CH solution and DW rises up to 12.72. These levels were assessed by a pH meter in our previous unpublished study on the properties of CH nanoparticles. Concerning the buffering capacity of dentin, it is suggested to assess the pH level in dentinal tubules in different time periods in future studies.

Also for a thorough assessment of the antimicrobial activity it is suggested to evaluate the antimicrobial effect of NCH on other bacterial species involved in root canal infection.

## Conclusion

Calcium hydroxide nanoparticles have superior antimicrobial activity against *E. faecalis* compared to conventional calcium hydroxide in culture media as well as dentinal tubules.
